# Catestatin, vasostatin, cortisol, temperature, heart rate, respiratory rate, scores of the short form of the Glasgow composite measure pain scale and visual analog scale for stress and pain behavior in dogs before and after ovariohysterectomy

**DOI:** 10.1186/s13104-016-2193-1

**Published:** 2016-08-02

**Authors:** Thanikul Srithunyarat, Odd V. Höglund, Ragnvi Hagman, Ulf Olsson, Mats Stridsberg, Anne-Sofie Lagerstedt, Ann Pettersson

**Affiliations:** 1 Department of Clinical Sciences, Swedish University of Agricultural Sciences, Box 7054, 75007 Uppsala, Sweden; 2 Department of Surgery and Theriogenology, Faculty of Veterinary Medicine, Khon Kaen University, Khon Kaen, 40002 Thailand; 3 Unit of Applied Statistics and Mathematics, Swedish University of Agricultural Sciences, Box 7013, 75007 Uppsala, Sweden; 4 Department of Medical Sciences, Uppsala University, 75185 Uppsala, Sweden

**Keywords:** Catestatin, Chromogranin A, Cortisol, Dog, Ovariohysterectomy, Pain, Short form of the Glasgow composite measure pain scale, Stress, Vasostatin, Visual analog scale

## Abstract

**Background:**

The stress reaction induced by surgery and associated pain may be detrimental for patient recovery and should be minimized. The neuropeptide chromogranin A (CGA) has shown promise as a sensitive biomarker for stress in humans. Little is known about CGA and its derived peptides, catestatin (CST) and vasostatin (VS), in dogs undergoing surgery. The objectives of this study were to investigate and compare concentrations of CGA epitopes CST and VS, cortisol, body temperature, heart rate, respiratory rate, scores of the short form of the Glasgow composite measure pain scale (CMPS-SF) and visual analog scales (VAS) for stress and pain behavior in dogs before and after ovariohysterectomy.

**Methods:**

Thirty healthy privately owned female dogs admitted for elective ovariohysterectomy were included. Physical examination, CMPS-SF, pain behavior VAS, and stress behavior VAS were recorded and saliva and blood samples were collected before surgery, 3 h after extubation, and once at recall 7–15 days after surgery. Dogs were premedicated with morphine and received carprofen as analgesia for 7 days during the postoperative period.

**Results:**

At 3 h after extubation, CMPS-SF and pain behavior VAS scores had increased (*p* < 0.0001) and stress behavior VAS scores, temperature, respiratory rate (*p* < 0.0001), plasma CST concentrations (*p* = 0.002) had decreased significantly compared to before surgery. No significant differences were observed in the subjective and physiological parameters between before surgery and at recall, but plasma CST (*p* = 0.04) and serum cortisol (*p* = 0.009) were significantly lower at recall. Plasma VS, saliva CST, and heart rate did not differ significantly at any observed time.

**Conclusion:**

Study parameters for evaluating surgery-induced stress and pain changed in dogs subjected to ovariohysterectomy. To further evaluate CST and VS usefulness as pain biomarkers, studies on dogs in acute painful situations are warranted.

## Background

Animals as well as humans may react to stress by stimulation of the sympatho-adrenal-medullary (SAM) axis and the hypothalamic–pituitary–adrenal (HPA) axis [1, 2]. Activation of these systems causes changes in physiological parameters such as heart and respiratory rate and cortisol, catecholamines, and neuropeptide secretion. Although essential for coping with acute changes in the body’s homeostasis, stress and particularly prolonged stress reactions can be detrimental [2–4].

Both acute and chronic pain, as well as surgery, can induce a stress response [5, 6]. The stress reaction induced by surgery is generally proportional to the degree of tissue trauma [7–9]. Postoperative stress and pain intensity can also be affected by other factors such as surgical skill and techniques, analgesic protocol, and complications [10–13]. Identifying and preventing prolonged pain and stress in surgically treated patients is therefore important for animal welfare and optimal recuperation [14, 15]. However, all currently available stress and pain assessment methods have shortcomings and new objective biomarkers would be of great value.

Chromogranin A (CGA) is a glycoprotein which is co-released with catecholamines when SAM is activated. Saliva and blood CGA have shown promise as useful stress biomarkers in humans, pigs, and dogs [16–18]. The bioactive epitopes of CGA, CGA17–38 (vasostatin) and CGA361–372 (catestatin), but not the intact CGA molecule, can be measured using radioimmunoassay in dogs [19]. Recently, reference ranges of catestatin (CST) and vasostatin (VS) in healthy dogs have been established [20]. In contrast to catecholamines, concentrations of CGA are rather stable during sample collection and storage [21]. In addition, concentrations of CST and VS have been shown to be unaffected by circadian, gender, age, and breed variation in dogs [20, 22, 23]. Few studies on CGA, CST, or VS have been performed in dogs [16, 19, 22–26] and no previous studies have compared CST and VS epitopes in healthy dogs prior to and in the peri- and postoperative periods in relation to both objective and subjective assessments of stress and pain.

Ovariohysterectomy (OHE) in healthy dogs is an elective surgical procedure which can be reasonably standardized. Cortisol concentrations, subjective scoring such as with the short form of Glasgow composite measure pain scale (CMPS-SF) and visual analog scale (VAS), and physiological parameters have previously been used in studies of the stress response induced by OHE in dogs [11, 13, 27–30]. The aims of this study were to investigate and compare concentrations of CST, VS, cortisol, body temperature, heart rate, respiratory rate, CMPS-SF score, pain behavior VAS and stress behavior VAS scores in healthy dogs before surgery, 3 h after postoperative extubation, and at recall 7–15 days after OHE.

## Methods

### Study design and ethical approval

This study was designed as a prospective clinical trial. The study was approved by Khon Kaen University (KKU) Ethical Legislation (AEKKU 26/2557). All dogs were treated in accordance to the routines at KKU Veterinary Teaching Hospital, Khon Kaen, Thailand. All dog owners were informed and gave their consent prior to participation.

### Dogs

Thirty privately-owned healthy female dogs appointed to perform elective OHE at KKU Veterinary Teaching Hospital during March to June 2015 were included in the study. Age, body weight, and body condition scores, from 10 different breeds including Chihuahua (n = 2), Thai ridgeback (n = 1), Thai bangkaew (n = 1), Pomeranian (n = 3), Shih tzu (n = 1), Maltese (n = 1), Siberian husky (n = 1), Labrador retriever (n = 1), Poodle (n = 1), and Mixed breeds (n = 18), are shown in Table 1. In all dogs, a standardized physical examination (mental status, general attitude, appetite, mucus membrane appearance, capillary refill time, rectal temperature, body weight, body condition score, hydration status, auscultation of heart and respiratory rate and sounds, abdominal palpation, musculoskeletal system palpation, lymph node palpation, hair and skin condition, mouth, ears, and eyes examination) and blood screening (hematology and blood biochemistry including creatinine, alanine aminotransferase, and total protein, and blood smears for *Dirofilaria immitis*, *Babesia canis*, *Hepatozoon canis*, *Ehrlichia canis, Trypanosoma evansi,* and *Anaplasma platys* screening) was performed prior to surgery. All dogs were healthy, as deemed by the history, standardized clinical examination, and preoperative blood screening and classified using American Society Anesthesiologists (ASA) Physical Status Classification System as ASA I. The OHE was performed within 3 weeks of the initial examinations. Food and water was withheld for 12 h before surgery.

**Table 1:** Age, body weight, and body condition score (BCS) in the included 30 healthy female dogs

Parameters	Mean ± SD	Range
Age (month)	28.4 ± 26.2	7–96
Body weight (kg)	11.6 ± 7.0	2.8–29.8
BCS (9 grade scale)	5.4 ± 1.1	3–7

### Anesthesia and surgery

Dogs were premedicated with 0.05 mg/kg acepromazine maleate (Combistress, Phoenix Pharmaceuticals, Antwerp, Belgium) and 0.4 mg/kg morphine (Morphine Sulfate injection, M&H manufacturing, Samutprakan, Thailand) intramuscularly. Fifteen minutes after premedication, an intravenous catheter was inserted into the distal cephalic vein and anesthesia was induced with intravenous administration of 1 % propofol with a dose range 3–6 mg/kg (Troypofol, Troikaa Pharmaceuticals, Uttarakhand, India) until tracheal intubation was possible. Anesthesia was maintained using inhaled isoflurane and pure oxygen in a rebreathing circuit (Matrx, VMS Anesthesia Machine, New York, USA). An intravenous infusion with Lactated Ringer solution (LRI, General Hospital Products Public, Pathumthani, Thailand) at a rate of 10 ml/kg/h was administered during surgery. Dogs were given prophylactic antibiotic treatment with 25 mg/kg cefazolin intravenously (Cefazol injection, Nida Pharma, Ayutthaya, Thailand). Oxygen saturation, pulse rate, respiratory rate, expiration carbon dioxide, and blood pressure were monitored continuously (Dash 4000, GE Medical System Information Technologies, Wisconsin, USA), and anesthetic depth was observed and monitored throughout the surgical procedure.

Ovariohysterectomy was performed aseptically by ventral midline approach. Dogs were placed on a heat pad during the procedure. The ovarian pedicle, broad ligament, and cervix were double ligated using chromic catgut (Catgut Chrom, Max suture, St. Vith, Belgium) and the uterus and ovaries were removed. The linea alba and subcutaneous tissues were closed using polyglactin 910 (Ethicon Coated Vicryl, Johnson & Johnson, Somerville, NJ, USA) and the skin was closed with polyamide (Supramid, Serag Wiessner, Nalia, Germany). All dogs were operated by the same experienced surgeon (TS).

### Recovery and recall

During recovery, dogs were observed in the recovery room until they were discharged on the same day as the surgery had been performed. At 3 h after extubation, 2.2 mg/kg carprofen (Rimadyl, Zoetis, Lincoln, USA) was administered subcutaneously. All dogs were treated with cephalexin, 25 mg/kg, (Lexporin, Nida Pharma, Ayutthaya, Thailand) and carprofen, 2.2 mg/kg, (Rimadyl, Zoetis, Lincoln, USA) orally twice daily for 7 days. Owners were telephone-interviewed on one occasion about their dog’s recovery status 3–5 days after surgery. Seven to fifteen days after surgery, dogs were recalled for evaluation of the wound healing and removal of skin sutures.

### Study protocol

The following procedures were performed in the same order in all dogs; standardized physical examination, CMPS-SF, overall pain behavior VAS (OP-VAS), and saliva and blood sample collection. Stress behavior using VAS (S-VAS) was scored separately for each individual saliva and blood sampling occasion. The described procedures were performed at three time points: before premedication, 3 h after extubation before carprofen administration, and once at the recall visit on day 7 to 15 after surgery.

### Subjective pain and stress assessments

**Fig. 1:**
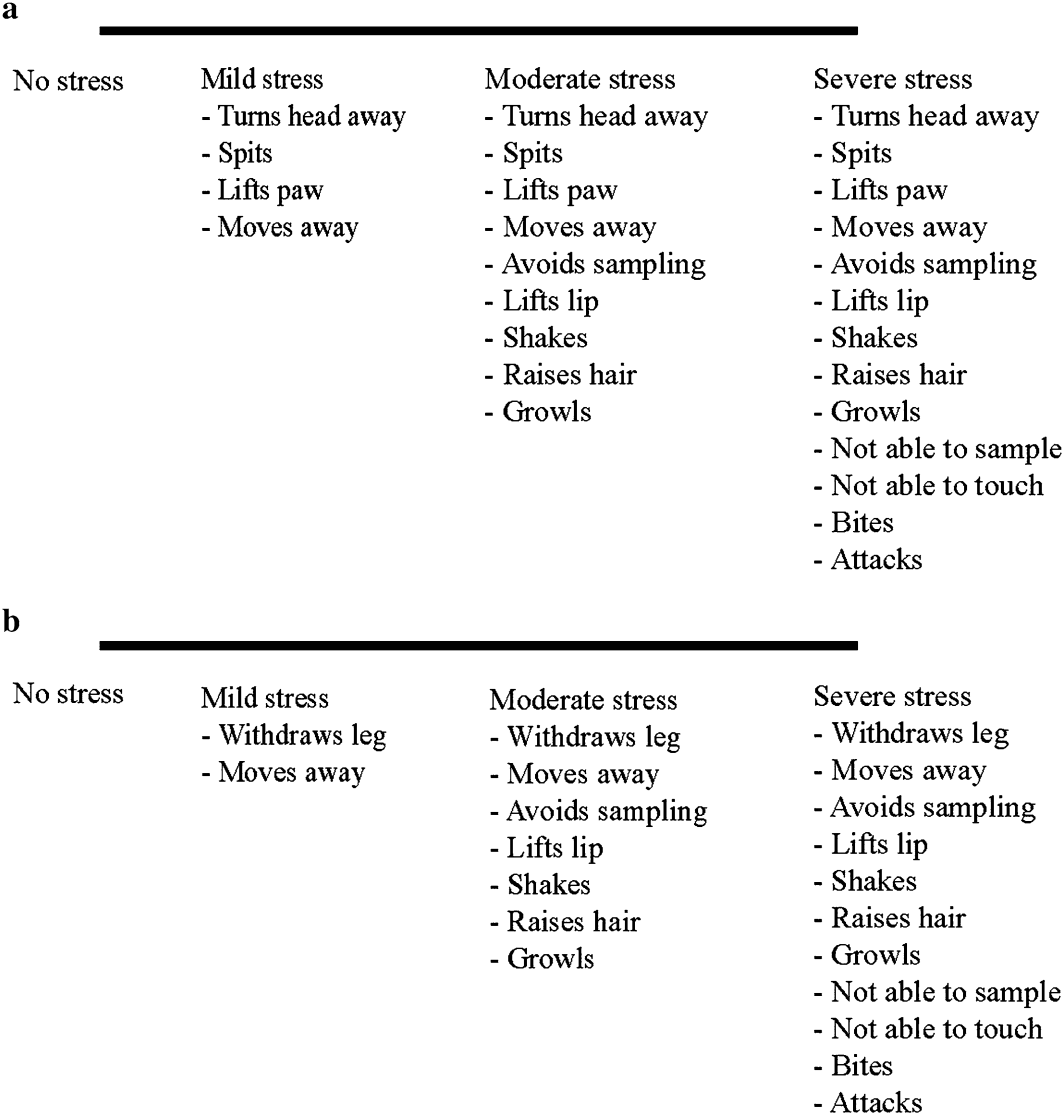
Stress behavior visual analog scale (S-VAS) criteria used during saliva and blood sampling [20]. **a** Criteria used during saliva sampling. **b** Criteria used during blood sampling Modified from [32]

The CMPS-SF was performed to score pain with a total score of 24 and a score ≥6/24 indicating the need for additional analgesia [31]. As stated previously, all dogs regardless of CMPS-SF scores, received carprofen analgesia after pain evaluation at 3 h after extubation. A 100-mm line VAS was used to score overall pain behavior VAS (OP-VAS), where the starting point corresponds to no pain and the end point to worst possible pain. The stress behavior VAS (S-VAS) assessed subjective stress by observed avoidance behaviors during saliva and blood sampling occasions. The pre-established criteria used for determination of S-VAS during saliva and blood sampling were modified from [32] see Fig. 1. In addition to taking all samples, the same observer (TS) performed all subjective pain and stress behavior assessments.

### Saliva and blood collection

Saliva and blood samples were collected prior to surgery (before premedication), 3 h after extubation, and at recall visit. Saliva samples were collected using swabs, sized 8 × 125 mm (SalivaBio Children’s swab, Salimetrics, PA, USA). A single swab was placed into the buccal cavity and the dog was allowed to chew the swab for 90 s. The swab was then transferred into a 17 × 100-mm swab storage tube (Swab storage tubes, Salimetrics, PA, USA) and centrifuged at 3000 rpm (1401 g) for 15 min. The saliva deposited was stored at −20 °C.

Blood samples were collected from the cephalic vein using butterfly needles (BD Vacutainer, Becton-Dickson, Plymouth, UK) into vacuum lithium heparin tubes and clot activator tubes (BD Vacutainer, Becton-Dickson, Plymouth, UK) and centrifuged at 3300 rpm (1695*g*) for 5 min. The obtained heparinized plasma and serum samples were frozen in cryotubes (Low Temperature Freezer Vials, VWR, Stockholm, Sweden) and stored at −20 °C. The order of sample collection (blood versus saliva) was randomized with an interval between saliva and blood sampling less than 5 min.

After clinical trials were completed, all samples were transported with temperature control and monitored to remain below −20 °C by a private transportation company (Temperature control, World Courier, Bangkok, Thailand) to the Swedish University of Agricultural Sciences (SLU) within 48 h. All samples were then frozen at −70 °C until analysis within a maximum of 7 months after collection.

### Catestatin and vasostatin analysis

Plasma and saliva samples were analyzed using rabbit antibodies against the human CGA amino acid sequence 17–38 for VS and sequence 361–372 for CST in duplicates using competitive radioimmunoassays at the Clinical Chemistry Laboratory, Uppsala University Hospital, Uppsala, Sweden as previously described [19]. The overall coefficient of variation (CV) was <10 %. For analysis, 300 µL saliva and 100 µL plasma were required. The saliva volumes obtained were insufficient for analysis of VS and only 21 of 90 saliva samples could be analyzed for CST.

### Cortisol analysis

Serum samples were analyzed for cortisol using solid-phase competitive chemiluminescent enzyme immunoassay (Immulite 2000, Siemens, Erlangen, Germany) at Clinical Chemistry Laboratory, SLU, Uppsala, Sweden. The overall CV was <5 %.

### Statistical analysis

Comparisons between the three time points (before surgery, 3 h after extubation, and at recall) were made using mixed linear models [33] with “dog” as a random factor.

In all analyses, residuals were checked for normality and homoscedasticity using diagnostic plots. Because the plasma VS values appeared skewed, this variable was log transformed (natural log) prior to analysis. Respiratory rate and heart rate for dogs with fast heart rate and panting was recorded as 200 per minute.

Pairwise comparisons were adjusted for multiplicity using Tukey’s method. All analyses were performed using the Mixed procedure in the SAS (2015) package [34]. The selected level of significance was *p* < 0.05.

## Results

The average (mean ± SD) dose of propofol used and surgical time from incision to skin closure was 4.1 ± 0.6 mg/kg and 30.8 ± 5.8 min. Based on the information reported by all owners during the postoperative telephone interview, there was no indication of complications in any of the dogs. The average recall day was 10 ± 1 days after surgery. Three dogs did not come for recall.

Concentrations of plasma CST, VS, saliva CST, and serum cortisol, temperature, heart rate, respiratory rate, and scores of the CMPS-SF, OP-VAS, saliva and blood sampling S-VAS scores for each time point are shown in Table 2. Although the average score of CMPS-SF was 4.4, eight dogs had CMPS-SF score ≥6/24 indicating pain, at 3 h after extubation.

**Table 2:** Measurement of subjective and objective assessments in dogs undergoing ovariohysterectomy

Parameters	Before surgery	Three hours after extubation	Recall
Plasma catestatin (nmol/L)^a^	0.76 ± 0.17	0.72 ± 0.16	0.74 ± 0.17
Plasma vasostatin (nmol/L)	1.12 ± 2.16	1.17 ± 2.49	1.45 ± 2.93
Saliva catestatin (nmol/L)	1.17 ± 0.48	0.74	1.09 ± 0.59
Serum cortisol^b^	174.6 ± 78.5	162.4 ± 88.4	122.8 ± 63.5
Temperature (°C)^c^	38.9 ± 0.4	37.5 ± 0.6	38.9 ± 0.4
Heart rate (beat per minute)	123.9 ± 30.6	109.5 ± 33.2	126.4 ± 32.0
Respiratory rate (time per minute)^c^	92.6 ± 63.3	28.5 ± 14.5	87.9 ± 62.7
The CMPS-SF (/24)^c^	0	4.4 ± 2.9	1.0 ± 1.3
OP-VAS (mm)^c^	0	28.8 ± 11.1	1.0 ± 1.9
Saliva sampling S-VAS (mm)^c^	41.6 ± 23.5	22.3 ± 16.3	35.4 ± 21.7
Blood sampling S-VAS (mm)^c^	37.7 ± 22.1	20.4 ± 20.2	28.8 ± 21.4

Data presented as mean ± SD in dogs undergoing ovariohysterectomy (n = 30)

Comparisons between groups were calculated using Tukey adjustment (*p* < 0.05)

The CMPS-SF, the short form of Glasgow composite measure pain scale; OP-VAS, overall pain behavior visual analog scale; and S-VAS, stress behavior visual analog scale

^a^ Levels at 3 h after extubation and at the recall visit differed significantly compared to before surgery and no significant difference was seen between 3 h after extubation and at recall

^b^ Levels at recall significantly differed from before surgery, no significant differences were found between before surgery and 3 h after extubation and between 3 h after extubation and at recall

^c^ Levels at 3 h after extubation significantly differed from before surgery and at recall where no significant difference was found between before surgery and at recall

**Fig. 2:**
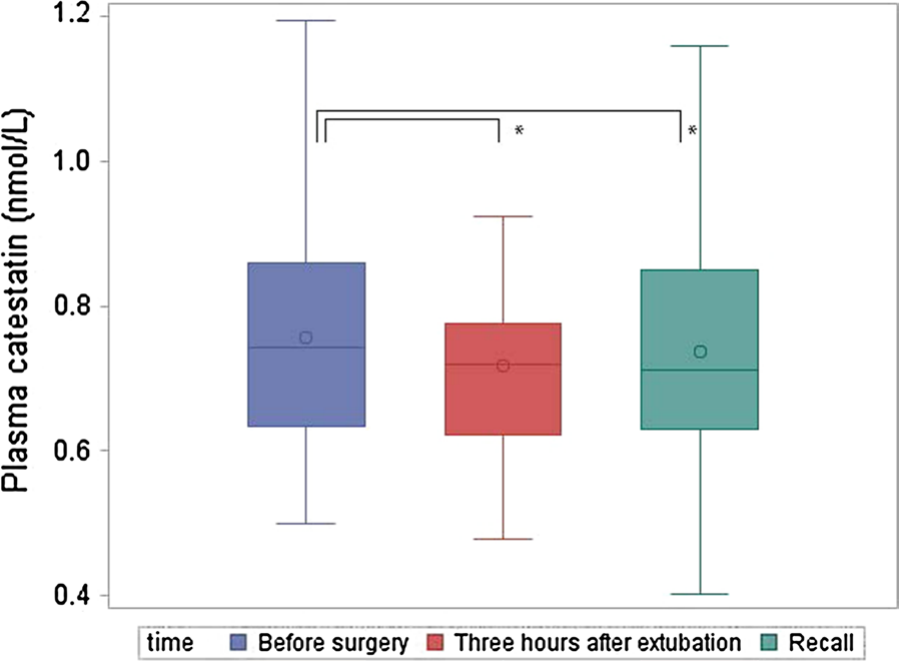
Plasma catestatin in 30 dogs undergoing ovariohysterectomy. *Asterisk* the plasma catestatin concentration was significantly decreased at 3 h after extubation (*p* = 0.002) and at recall (*p* = 0.04) compared to before surgery. No significant difference was seen between 3 h after extubation and recall (*p* = 0.56)

**Fig. 3:**
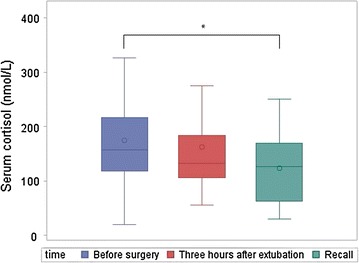
Serum cortisol in 30 dogs undergoing ovariohysterectomy. *Asterisk* serum cortisol concentration at recall was significantly decrease compared to before surgery (*p* = 0.009). No significant differences were seen between before surgery and 3 h after extubation (*p* = 0.73) and between 3 h after extubation and at recall (*p* = 0.06)

**Fig. 4:**
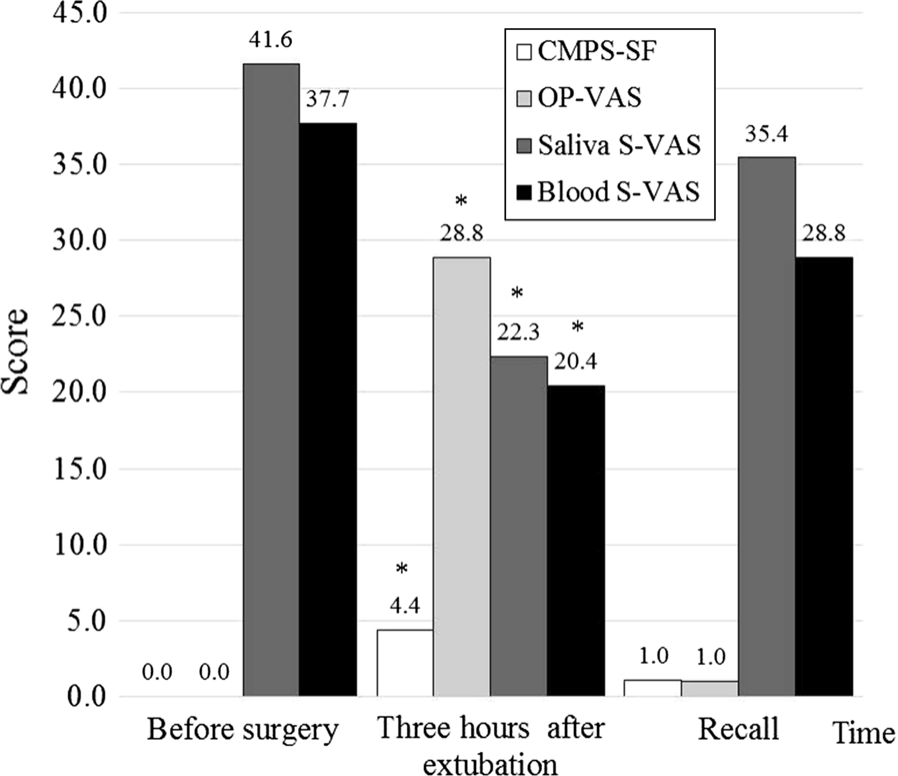
Subjective parameters in 30 dogs undergoing ovariohysterectomy. *Asterisk* All subjective parameters of short form of Glasgow composite measure pain scale (CMPS-SF), overall pain visual analog scale (OP-VAS), saliva sampling stress behavior visual analog scale (saliva S-VAS), and blood sampling stress behavior visual analog scale (blood S-VAS) significantly changed at 3 h after extubation compared to before surgery and at recall. No difference was found between before surgery and at recall

Plasma CST concentrations were significantly decreased 3 h after extubation (*p* = 0.002) and at the recall visit (*p* = 0.04) compared to before surgery (Fig. 2). Serum cortisol concentration at recall was significantly lower than before surgery (*p* = 0.009) as illustrated in Fig. 3. The CMPS-SF and OP-VAS were significantly increased at 3 h after extubation compared to before surgery and at recall (*p* < 0.0001) whereas saliva (*p* = 0.0003) and blood sampling S-VAS (*p* = 0.03), temperature (*p* < 0.0001), and respiratory rate (*p* < 0.0001) were significantly decreased (Fig. 4). Prior to surgery, the CMPS-SF and OP-VAS in all dogs were zero. Plasma VS, saliva CST, and heart rate did not significantly change in any of time points.

## Discussion

Chromogranin A is co-released with catecholamines in response to stress when the SAM axis is activated. Catecholamines can be used for monitoring stress both in humans and animals [13, 35], but have short half-lives and rapid degradation requiring rapid sample freezing which limit their use in a clinical setting [36]. Saliva and plasma CGA have been shown to be useful as stress biomarkers in both humans and animals [16–18]. In dogs with acute stress associated with insulin induced hypoglycemia, plasma CGA has been shown to correlate with plasma catecholamines and cortisol [16]. In contrast to catecholamines, CGA has a longer half-life, is stable for several repeated freeze-thawing cycles, and is therefore more suitable for use as a biomarker in clinical setting [21, 37]. In our study, CST and VS values reflect both the intact CGA molecule and the two respective degradation derived peptides. Plasma CST, both at 3 h after extubation and at recall, was significantly decreased compared to before surgery whereas plasma VS and saliva CST did not differ between the different sampling occasions. However, the concentrations of plasma CST in this study were within the reference ranges previously reported in healthy dogs accustomed to the sampling procedure [20]. The insufficient saliva volume obtained resulted in few analyzed saliva samples and therefore interpretation of saliva CST should be done cautiously. Although further studies are needed, the different values of CST and VS seen in our study may reflect different clearance rates of the bioactive peptides. Catestatin has an inhibitory role as a negative feedback for the release of catecholamines and CGA [38] which may contribute to the decreased concentrations of plasma CST in the early postoperative period and at recall. Levels of the CGA epitopes CST and VS in healthy dogs accustomed to the sampling procedures have recently been established [20]. In the present study, we investigated CST and VS and different established parameters for pain and stress monitoring in dogs undergoing OHE receiving appropriate pain medication. In order to further evaluate CST and VS usefulness as pain biomarkers, studies on dogs in acute painful situations are warranted.

Cortisol has been used as a stress and pain biomarker in both humans and animals [39–42]. Although circadian variation is still controversial, cortisol is secreted episodically [43–45]. Serum cortisol decreased significantly at recall but not at 3 h after extubation compared to before surgery. A secretion of cortisol occurs within 30 min after activation of the HPA axis and the half-life is approximately 66 min [46]. In previous studies of dogs undergoing OHE, the surgery induced a stress response as indicated by hypersecretion of cortisol upon removal of the ovaries [26, 47]. Moreover, studies on different analgesic protocols for dogs undergoing OHE have shown that circulating cortisol levels are elevated at 30 min after induction of anesthesia until 60–240 min after extubation depending on surgical technique, anesthesia, and analgesic protocol [11–13, 28]. Although intraoperative cortisol concentrations were not measured in the present study, the results at 3 h after extubation were in agreement with previous studies, using a similar analgesic protocol, in which cortisol had returned to the preoperative values within 2 h after extubation [11, 13]. In our study, at the recall visit for removal of skin sutures, serum cortisol was significantly decreased compared to before surgery. Many different stimuli can induce stress reactions, including fear and psychological anxiety which is often the case when animals are brought to a clinical practice. This phenomenon is commonly known as the white coat effect and has been shown to lead to increase secretion of cortisol [48–50]. The higher levels of cortisol and CST seen before surgery in the present study could possibly be due to the white coat effect with less response at recall when the dogs were more accustomed to the animal hospital. However, although VAS needs to be interpreted cautiously, there was no significant difference in stress behavior VAS scores between before surgery and at recall indicating that dogs may have experienced similar psychological stress at the two time points. After prolonged HPA stimulation, as in chronic pain and stress disorders, cortisol has been shown to decrease in both humans and animals [45, 48, 51, 52]. It can therefore not be excluded that the decreased cortisol and CST levels seen at recall might indicate an underlying persistent stress response after the OHE procedure [53].

Physiological parameters can be affected by several factors. In our study, temperature and respiratory rate significantly decreased during anesthetic recovery and returned to levels similar to before surgery at recall. The high breathing rate observed in dogs prior to surgery and at recall was due to panting. Heart rate did not change significantly although it decreased at 3 h after extubation, similarly to temperature and respiratory rate. The temperature and respiratory rate decrease in our study is probably an effect of premedication and anesthesia [54].

The findings in this study, using subjective pain and stress behavior assessments, showed that at 3 h after extubation, dogs experienced pain and their avoidance ability was reduced. Subjective pain and stress assessments are simple, quick, and can be useful for evaluating acute pain in a clinical setting. Pain is subjective and the perception of pain can differ between individuals. Although VAS pain scores have been widely used in both humans and dogs, they should be interpreted with caution [55–57]. Even in humans, where VAS for monitoring pain is self-evaluated, the same individual’s response to a standardized pain stimuli may vary [56]. In dogs, the CMPS-SF has been validated for assessing acute pain [31]. Both CMPS-SF and VAS, however, require a trained single and preferably blinded observer [58] to reduce bias and inter-observer variation. Until now, no validated subjective pain score for chronic pain in dogs has been presented, which illustrates the need for new pain biomarkers.

This study was performed in clinical veterinary practice and therefore several limitations must be taken into account. Ideally, to exclude the effect of anesthesia and analgesia, a sham group (anesthesia only, or minor surgery) should have been included, but this was not possible for ethical reasons. Prophylactic antibiotic treatment is controversial; however, it is the routine treatment at KKU Veterinary Teaching Hospital and, therefore, included in the study protocol. It would have been preferable to take samples at the same time of day in all patients; however, this was not possible for practical reasons. Sedation can influence subjective assessments and behaviors during recovery from anesthesia [55], and sedative scores were not measured in this study. Ideally, the subjective assessments should also have been be blinded.

## Conclusions

Concentrations of plasma CST, serum cortisol, body temperature, respiratory rate, scores of CMPS-SF and stress and pain behavior VAS for evaluating surgical stress and pain changed in dogs subjected to ovariohysterectomy. To further evaluate CST and VS usefulness as pain biomarkers, studies on dogs in acute painful situations are warranted.

## Abbreviations

CGA: chromogranin A
CMPS: Glasgow composite measure pain scale
CMPS-SF: short form of the Glasgow composite measure pain scale
CST: catestatin
CV: coefficient of variation
OP-VAS: overall pain behavior visual analog scale
RIA: radioimmunoassay
RPM: revolutions per minute
S-VAS: stress behavior visual analog scale
VS: vasostatin
VAS: visual analog scale
